# Treatment of hepatitis C in HIV patients in the new DAA era

**DOI:** 10.1186/1471-2334-14-S2-S16

**Published:** 2014-05-23

**Authors:** Vicente Soriano

**Affiliations:** 1 Department of Infectious Diseases, Hospital Carlos III, Madrid, Spain

## 

Of 35 million people currently living with HIV worldwide, around 4 million (12%) have chronic hepatitis C. Although intravenous drug users are overepresented within the HIV-HCV coinfected population, recent outbreaks of acute hepatitis C among homosexual men highlight that HCV can also be transmitted efficiently by sex. Progression to cirrhosis, liver insufficiency and hepatocellular carcinoma occurs in coinfected patients more rapidly than in HCV-monoinfected individuals. Therefore, antiviral therapy for hepatitis C has become a priority in this population.

Classical therapy based on interferon provided low rates of HCV cure in the HIV setting. Although the advent of first generation direct-acting antivirals boceprevir and telaprevir increased sustained virological response rates, it was at the cost of a high rate of side effects and significant drug interactions with antiretrovirals.

The recent approval of sofosbuvir and simeprevir has revolutionized the field, as these agents in combination with peginterferon-ribavirin provide high rates of cure without adding noticeable toxicity and drug interactions and with shorter treatment duration. Moreover, sofosbuvir plus ribavirin alone provides cure to most patients with HCV genotypes 2 and 3. On the other hand, the combination of sofosbuvir plus simeprevir may cure most patients infected with HCV genotypes 1 or 4 ineligible for interferon.

All these unprecedent successes, however, are currently challenged by the very high cost of these medications. Unless their price goes down or new oral anti-HCV agents expected to be approved soon (i.e., daclatasvir and/or faldaprevir) are marketed at lower prices, it is unlikely a rapid widespread use of antiviral therapy for chronic hepatitis C in coinfected patients. The risk is further marginalization of groups and persons.

